# RCoV19: A One-stop Hub for SARS-CoV-2 Genome Data Integration, Variant Monitoring, and Risk Pre-warning

**DOI:** 10.1016/j.gpb.2023.10.004

**Published:** 2023-10-26

**Authors:** Cuiping Li, Lina Ma, Dong Zou, Rongqin Zhang, Xue Bai, Lun Li, Gangao Wu, Tianhao Huang, Wei Zhao, Enhui Jin, Yiming Bao, Shuhui Song

**Affiliations:** 1 National Genomics Data Center, Beijing Institute of Genomics, Chinese Academy of Sciences and China National Center for Bioinformation, Beijing 100101, China; 2 CAS Key Laboratory of Genome Sciences and Information, Beijing Institute of Genomics, Chinese Academy of Sciences and China National Center for Bioinformation, Beijing 100101, China; 3 University of Chinese Academy of Sciences, Beijing 100049, China; 4 Sino-Danish College, University of Chinese Academy of Sciences, Beijing 100049, China

**Keywords:** SARS-CoV-2, Mutation, Variants, Surveillance, Pre-warning

## Abstract

The Resource for Coronavirus 2019 (RCoV19) is an open-access information resource dedicated to providing valuable data on the genomes, **mutations**, and **variants** of the severe acute respiratory syndrome coronavirus 2 (**SARS-CoV-2**). In this updated implementation of RCoV19, we have made significant improvements and advancements over the previous version. Firstly, we have implemented a highly refined genome data curation model. This model now features an automated integration pipeline and optimized curation rules, enabling efficient daily updates of data in RCoV19. Secondly, we have developed a global and regional lineage evolution monitoring platform, alongside an outbreak risk **pre-warning** system. These additions provide a comprehensive understanding of SARS-CoV-2 evolution and transmission patterns, enabling better preparedness and response strategies. Thirdly, we have developed a powerful interactive mutation spectrum comparison module. This module allows users to compare and analyze mutation patterns, assisting in the detection of potential new lineages. Furthermore, we have incorporated a comprehensive knowledgebase on mutation effects. This knowledgebase serves as a valuable resource for retrieving information on the functional implications of specific mutations. In summary, RCoV19 serves as a vital scientific resource, providing access to valuable data, relevant information, and technical support in the global fight against COVID-19. The complete contents of RCoV19 are available to the public at https://ngdc.cncb.ac.cn/ncov/.

## Introduction

SARS-CoV-2 is responsible for the COVID-19 pandemic, and continues to evolve and spread to threat public health worldwide. Genome data play a crucial role in understanding mutations (refers to an actual nucleotide or amino acid change in a viral genome) and supporting the design of candidate vaccines. While there are various data deposition repositories available, such as GISAID EpiCoV^TM^ [1], GenBank [2], [3], and GenBase (https://ngdc.cncb.ac.cn/genbase/), none of them encompass all worldwide genome data, and redundancies exist among these repositories. Therefore, the need for a comprehensive SARS-CoV-2 database arises to integrate genome data, monitor evolution, and provide pre-warning for high-risk variants. Such a database is essential to comprehend the ongoing pandemic and facilitate timely adjustments to public health interventions.

With millions of genome sequences now available, several platforms have been developed to track SARS-CoV-2 mutations. These platforms, including COVID-19 CG [4], Outbreak [5], CoV-Spectrum [6], CovMT [7], Regeneron COVID-19 dashboard (https://covid19dashboard.regeneron.com/), and ViruClust [8], enable tracking of mutations by sampling location or date of interest, and known variants globally. Among them, CovMT utilizes mutation fingerprints to facilitate geographic tracking and includes disease severity information, and ViruClust enables comparison of mutations from two unrelated locations in terms of hierarchy. Besides, Vcorn database allows for global and domestic data search on COVID-19 infections and mutations in *S* gene via correlation network analysis [9]. VarEPS [10] assesses the risk level of mutations and variants based on their transmissibility and affinity to neutralizing antibodies. Additionally, databases like CoV-RDB [11], [12] and COG-UK-ME [13] have compiled mutations associated with reduced susceptibility to various factors, such as clinical stage SARS-CoV-2 Spike monoclonal antibody (mAb), RNA-dependent RNA polymerase (RdRP) inhibitor, 3C-like protease (3CLpro) inhibitor, or mutations on T cell epitope. However, despite these significant efforts, there are limitations in terms of efficiency and comprehensiveness. Most of these platforms and databases only focus on specific aspects of SARS-CoV-2 monitoring or prevention (Table S1).

Furthermore, numerous important mutations affecting transmissibility, infectivity, or expression are scattered throughout published literature. Consequently, there is an urgent need to build an integrated and comprehensive system that encompasses “data–information–knowledge–application”. This system should provide real-time services for sequence monitoring, evolution tracking, and pre-warning of high-risk variants.

**Figure 1:**
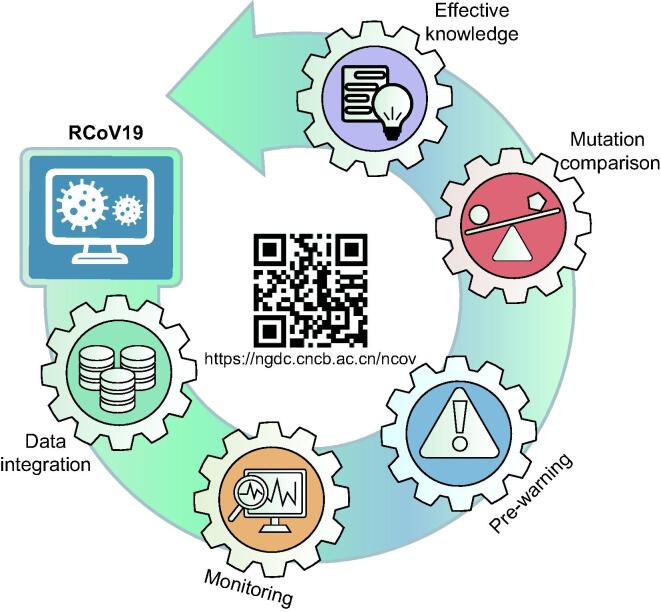
**Logical architecture diagram of RCoV19 database**

RCoV19, previously known as 2019-nCoVR [14], [15], is an open-access information resource for SARS-CoV-2. It has been available online and has already provided data services to over 3.2 million visitors from 182 countries/regions worldwide, with more than 14 billion data downloads in total. In this updated release of RCoV19, significant improvements have been made in data curation, integration, sequence growth and lineage evolution surveillance, and mutation comparisons of sequences and lineages. Additionally, a weekly report on potentially high-risk haplotypes (a distinct virus genome sequence) and variants (a viral genome that may contain one or more mutations which may affect virus’s properties) is provided by considering genetic mutation effects and haplotype network features [16], [17]. Furthermore, RCoV19 curates an integrated knowledge of mutation effects from literature and databases, offering critical insights into virus evolution, immune escape, and medical countermeasures. Ultimately, RCoV19 establishes a one-stop hub for SARS-CoV-2 genome data integration and variant monitoring, as illustrated in Figure 1.

## Database content and features

### Efficient integration and retrieval of worldwide SARS-CoV-2 genome data

**Figure 2:**
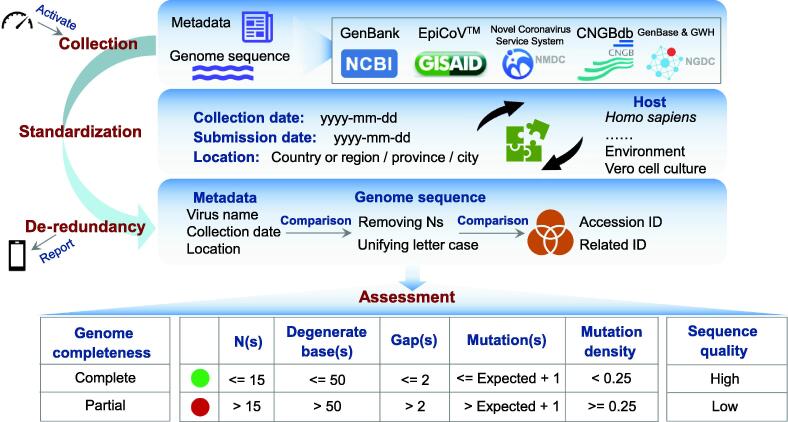
**Framework of genome data curation model for SARS-CoV-2** RCoV19 integrates genome data from different repositories and provides value-added curations. It collects metadata and genome sequences from different resources, standardizes metadata, and performs de-redundancy processing based on metadata and sequence comparisons. These steps have been chained together as one workflow, which is activated automatically every day and sends the integration statistics to mobile phone client at the end. After integration, RCoV19 performs a series of assessments; it determines completeness of the protein-coding region, assesses sequence quality in five aspects, and defines high-quality sequences. We consider a sequence to be of high quality if it could pass quality control for both Ns (<= 15) and degenerate bases (<= 50). Otherwise, it is of low quality.

RCoV19 is an extensive data resource for SARS-CoV-2 that collects genome data from multiple repositories, performs de-redundancy processing, and assesses sequence quality to ensure a comprehensive and curated collection of worldwide genomes (Figure 2). The resource incorporates data from EpiCoV^TM^ [1], GenBank [2], [3], CNGBdb [18], and Novel Coronavirus Service System of NMDC [19], and has included data from GenBase since the beginning of 2023. To eliminate redundancies that have been submitted to different data sources, RCoV19 identifies identical genomes across different sources and cross-references related accession IDs. In the de-redundancy processing, we place a significant emphasis on comparing key metadata such as virus name (specifically the isolate name), sampling date, and location. These comparisons serve as the primary method for identifying identical sequences across different databases. To facilitate this process, we standardize these metadata beforehand and employ manual curation to identify additional identical sequences with similar but not completely consistent metadata. In addition, we take certain standardization measures such as unifying the letter case of genome sequences, removing Ns from the entire sequence, and calculating the MD5 codes for the standardized sequences. By comparing these MD5 codes, we are able to identify more identical sequences from different data sources. Therefore, we detect sequences that are deposited to multiple sources by matching metadata (isolate name, sampling date, and location) or both metadata and sequences. Notably, different from the statistics reported at the early stage of COVID-19 where all overlaps were found between GISAID and other sources [20], EpiCoV^TM^ doesn’t cover all the sequences, but has achieved coverage of 96% of sequences worldwide. It has an overlap of 91.3% with GenBank sequences, while 56.7% of EpiCoV^TM^ sequences are unique (Figure S1). In addition, RCoV19 determines completeness of the protein-coding region, evaluates sequences in five aspects (Ns, degenerate bases, gaps, mutations, and mutation density), and defines high-quality sequences based on Ns and degenerate bases. These processes enable RCoV19 to provide a comprehensive and reliable list of SARS-CoV-2 genomes for global monitoring and pre-warning purposes.

In the new version, the SARS-CoV-2 genome data curation model has been significantly enhanced with an automated integration pipeline and optimized curation rules (Figure 2), ensuring efficient daily updates in RCoV19. The automated pipeline, activated by a timer every day, collects genome data from those repositories through the Chrome Browser on Linux, standardizes genome metadata, and performs de-redundancy processing. This automated approach improves efficiency compared to semi-automated methods and enables regular and constant updates. To ensure the reliability of the automated approach in data collection and curation, we have implemented a series of measures. Firstly, the integration statistics for each step will be shared with the mobile phone client to confirm successful data download and processing. Secondly, we employ strict criteria to identify redundant sequences during the automated processing stage. Thirdly, we incorporate manual curation to detect additional redundant sequences. Lastly, considering that SARS-CoV-2 genome data are always updated in different data sources [21], we examine the sequence and metadata changes to ensure data in RCoV19 remain up-to-date. Furthermore, curation rules have also been optimized to achieve more accurate de-redundancy, by comparing genome sequences (with the removal of Ns and uniform letter cases) in addition to key metadata (virus name, sampling date, and location). The curation rule for assessing abnormally high mutations has been improved as well. The expected number of mutations for each sequence is now calculated based on its sampling date and empirical mutation rate [22], rather than relying on a fixed value. This approach provides a more realistic assessment. If the observed number of mutations exceeds the expected number, the genome sequence is highlighted with a red dot, indicating the need for further investigation into sequencing quality issues.

With the automated integration pipeline and optimized curation rules, RCoV19 accommodated a total of 16,119,080 non-redundant genome sequences from 193 countries/regions as of June 10, 2023. A comprehensive and up-to-date list of all released SARS-CoV-2 genome metadata can be freely accessed and downloaded by users at https://bigd.big.ac.cn/ncov/release_genome. The majority of these genomes are contributed by countries such as the United States (31.6%), United Kingdom (19.3%), Germany (5.9%), France (4.4%), Denmark (4.0%), Japan (3.8%), and Canada (3.4%). Among the released human-derived genome sequences (16,103,219), 87.7% are complete, and 47.0% are both complete and high-quality. Additionally, RCoV19 offers the service of collapsing identical sequences, resulting in a total of 5,832,804 unique sequences (1:1.3) among the complete and high-quality human-derived genome sequences, and 13,762,271 unique sequences (1:1.2) among all released genomes, highlighting the rapid evolution and high diversity of SARS-CoV-2 genomes.

To facilitate fast and customized retrieval of SARS-CoV-2 genomes from this vast collection, RCoV19 has developed an advanced search module at https://ngdc.cncb.ac.cn/ncov/genome/search. Users can query items by accession ID, Pango lineage, World Health Organization (WHO) variant label, country/region, host, nucleotide completeness, quality assessment, database resource, sampling date, and sequence length range. The search results are complemented by statistics displayed on the right side of the search page, showcasing distributions in nucleotide completeness, sequence quality, data source, WHO variant label, lineage, country/region, and host. Furthermore, all filtered results can be easily downloaded to support downstream analysis.

### Timely monitoring of sequence growth and lineage evolution

With the rapid accumulation of SARS-CoV-2 genome sequences, the emergence of new lineages in specific regions or the whole world has become increasingly prevalent. To enhance our understanding of SARS-CoV-2 evolution and transmission characteristics, we have developed specific modules (https://ngdc.cncb.ac.cn/ncov/monitoring/global) for monitoring global and regional sequence growth and lineage evolution.

**Figure 3:**
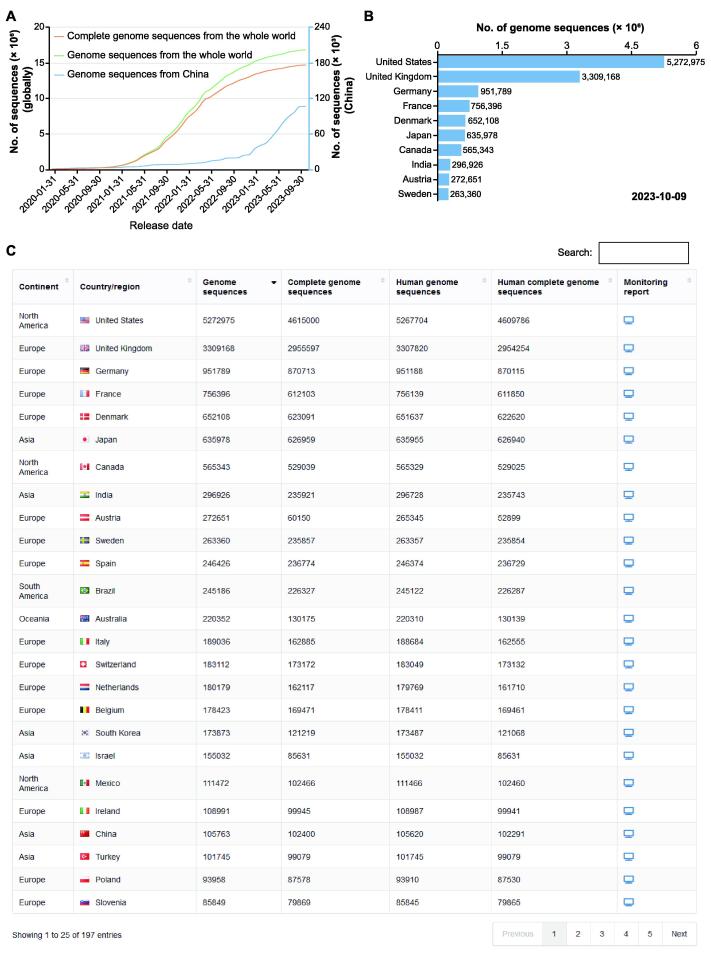
**The monitoring platform of SARS-CoV-2 sequence growth globally and regionally** **A.** The dynamic growth curves of globally and China released SARS-CoV-2 genome sequences, and globally released complete genome sequences as of October 9, 2023. The vertical coordinate with the blue hue on the right-hand side represents China’s data. **B.** A bar chart shows the top 10 countries with the most public released genome sequences as of October 9, 2023. **C.** A tabular table shows the statistics of sequences in country/region.

Sequence growth serves as an indicator of a country’s monitoring capability and level. By examining the cumulative curve of genome sequence growth based on release dates, we can identify three distinct periods: slow growth (January 2020 to January 2021), fast growth (February 2021 to May 2022), and relatively slow growth (June 2022 to October 2023) (Figure 3A). We dynamically display the genome sequence numbers for the top 10 countries each month to visualize their contributions (Figure 3B). Moreover, we organize sequence numbers for each country/region in a tabular format to provide more detailed data (Figure 3C). For example, as of October 9, 2023, a total of 105,763 genome sequences have been released for China, with an average release rate of hundreds of sequences per month in 2023.

**Figure 4:**
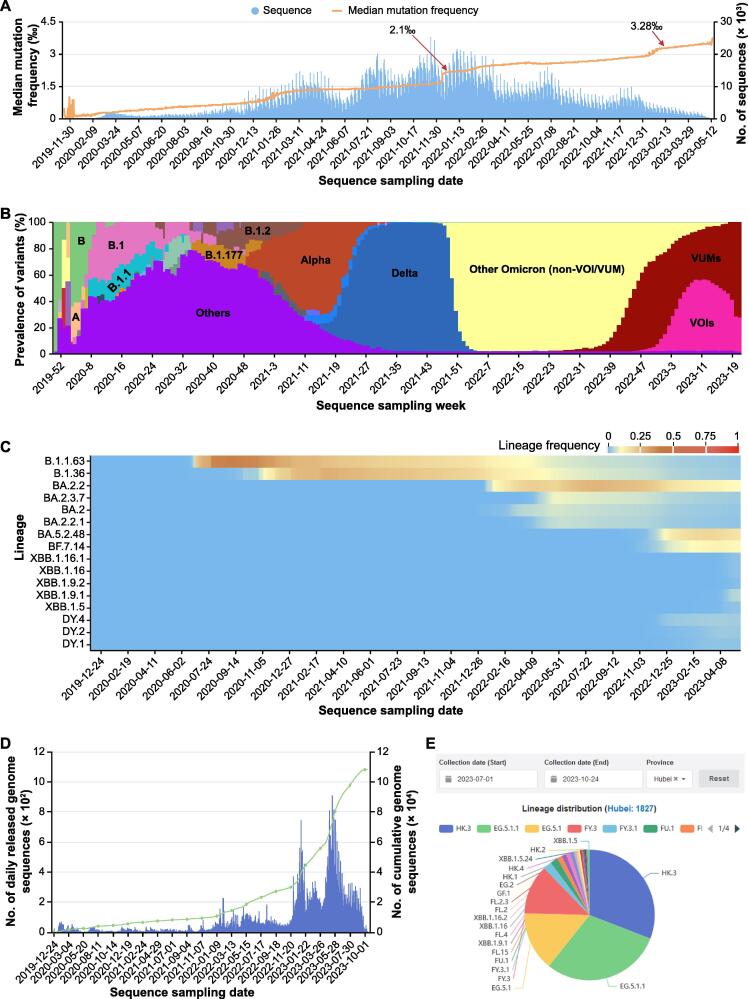
**Monitoring of SARS-CoV-2 lineage evolution globally and regionally** **A.** Number of released genome sequences and the median mutation frequency along sequence sampling date. The mutation frequency is calculated by dividing the total mutation of each sequence by the genome length. **B.** Stacking diagram showing the proportion of top 3 prevalent Pango lineages or WHO-defined variants per week. VUMs are currently circulating variants under monitoring that include BA.2.75, CH.1.1, XBB*, XBB.1.9.1, XBB.1.9.2, and XBB.2.3 Pango lineages as of 19 June 2023. Meanwhile, VOIs are currently circulating variants of interest that include XBB.1.5 and XBB.1.16 Pango lineages as of 27 June 2023. **C.** Heatmap showing the frequency of the cumulative sequences for randomly selected lineages in China. **D.** Distribution of the number of genome sequences released in China along sequence sampling date. The blue vertical line represents the number of genome sequences released daily in China, and the green line represents the dynamic accumulation of SARS-CoV-2 genome sequences released in China. **E.** Pie chart showing the lineage proportions in Hubei Province in China from July 1 to October 24 in 2023. VUM, variant under monitoring; VOI, variant of interest.

As SARS-CoV-2 spreads, mutations constantly occur and accumulate, leading to the emergence of new lineages and variants. To monitor mutation rates, we calculate the mutation frequency (mutation numbers / genome length) for each complete and high-quality genome and plot the daily median mutation frequency as a curve (Figure 4A). By observing the slope of curve growth, it is facilitated to timely monitor signals indicating accelerated mutation. For instance, the median mutation frequency rapidly increased to 2.1‰ in mid-December 2021 due to the rapid spread of Omicron variant and reached 3.28‰ in March 2023 due to the spread of XBB.1.5 variant. As sequences with similar mutation spectra are always classified into a Pango lineage [23] or named as a WHO-defined variant (https://www.who.int/news/item/31-05-2021-who-announces-simple-easy-to-say-labels-for-sars-cov-2-variants-of-interest-and-concern), we display the weekly sequence proportion for each lineage or variant. To highlight the main lineages or variants that are currently or previously popular, we interactively display only the top 3 Pango lineages or WHO-defined variants with the most genome sequences (Figure 4B). Additionally, the genome sequence proportion for each lineage is further represented in a heatmap (Figure 4C), providing informative insights into lineage trends over time. Taking China as an example, it experienced a wave of COVID-19 infections from late 2022 to early 2023. We use a histogram to display the distribution of the number of sequences released daily in China, and we also provide a pie chart to monitor the prevalence of SARS-CoV-2 transmission at the provincial level in China (Figure 4D and E).

### Pre-warning of potential high-risk haplotypes and lineages

**Figure 5:**
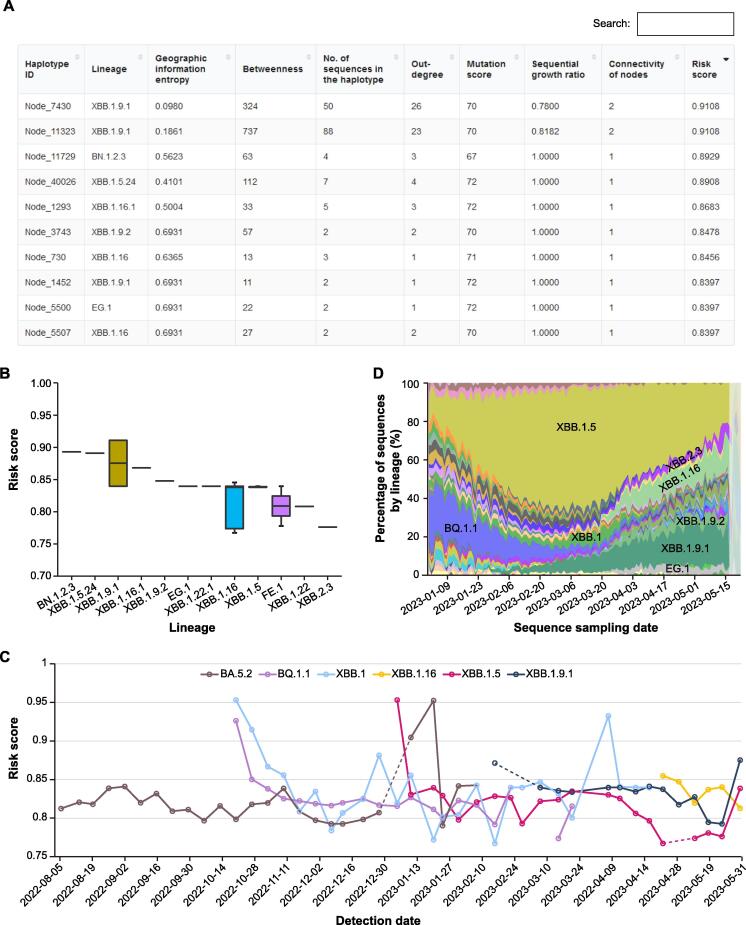
**Pre-warning of potential high-risk haplotypes and lineages** **A.** A screenshot of the tabular table for all haplotypes with values of haplotype network features and their risk scores predicted on May 31, 2023. **B.** Boxplot showing the predicated risk scores for all haplotypes of higher-risk lineages (20 at most). As of May 31, 2023, 12 lineages have been predicted as potential high-risk lineages. **C.** Distribution of the historical risk scores for user selected lineages. **D.** Genomic prevalence of lineages based on sequence sampling date.

Early and accurate detection of potential high-risk SARS-CoV-2 haplotypes or lineages is a shared challenge for the scientific community in combating the virus. Leveraging the vast amount of genome sequences, we have developed a machine learning model called HiRiskPredictor [16] to predict potential high-risk haplotypes and update these predictions weekly in RCoV19 (https://ngdc.cncb.ac.cn/ncov/monitoring/risk). For each haplotype, a risk score ranging from 0 to 1 is calculated based on the available sequences at that time. Haplotypes with higher risk scores (> 0.5) are identified as potential high-risk haplotypes. A tabular table (Figure 5A) organizes the risk score, associated lineage, and transmission-related values (*e.g.*, geographic entropy and betweenness) for each high-risk haplotype. Users can quickly search for specific haplotypes or lineages using different keywords, or sort the table by ‘Risk score’ to identify haplotypes with the highest risk scores. Additionally, a boxplot displays those higher risk lineages (20 at most), ranked in descending order based on the median risk scores of all associated haplotypes. Figure 5B illustrates the prediction of 12 potential high-risk lineages as of May 31, 2023, with BN.1.2.3, XBB.1.5.24, XBB.1.9.1, XBB.1.16.1, and XBB.1.9.2 identified as the top 5 lineages. Importantly, the weekly predicted risk scores for all lineages are recorded, allowing users to track historical predictions, to detect new warning lineages, and to understand their development trends (Figure 5C). Furthermore, the lineage prevalence, represented by the genome sequence proportion, is plotted to visualize global changes in epidemic variants (Figure 5D). For example, the dominant lineage XBB.1.5 accounts for 20% of all Omicron lineages but is gradually diminishing and being replaced by XBB.1.9.1.

These visualization modules provided by RCoV19 empower users to identify potential high-risk haplotypes and track the prevalence and evolution of lineages, contributing to early warning systems and informed decision-making in the fight against SARS-CoV-2.

### Mutation spectrum comparison between selected lineages or sequences

To facilitate the analysis of mutation spectra and comparisons between different lineages and sequences of SARS-CoV-2, we have developed two interactive modules for users to explore mutation distributions and construct mutation maps at lineage level or sequence level.

**Figure 6:**
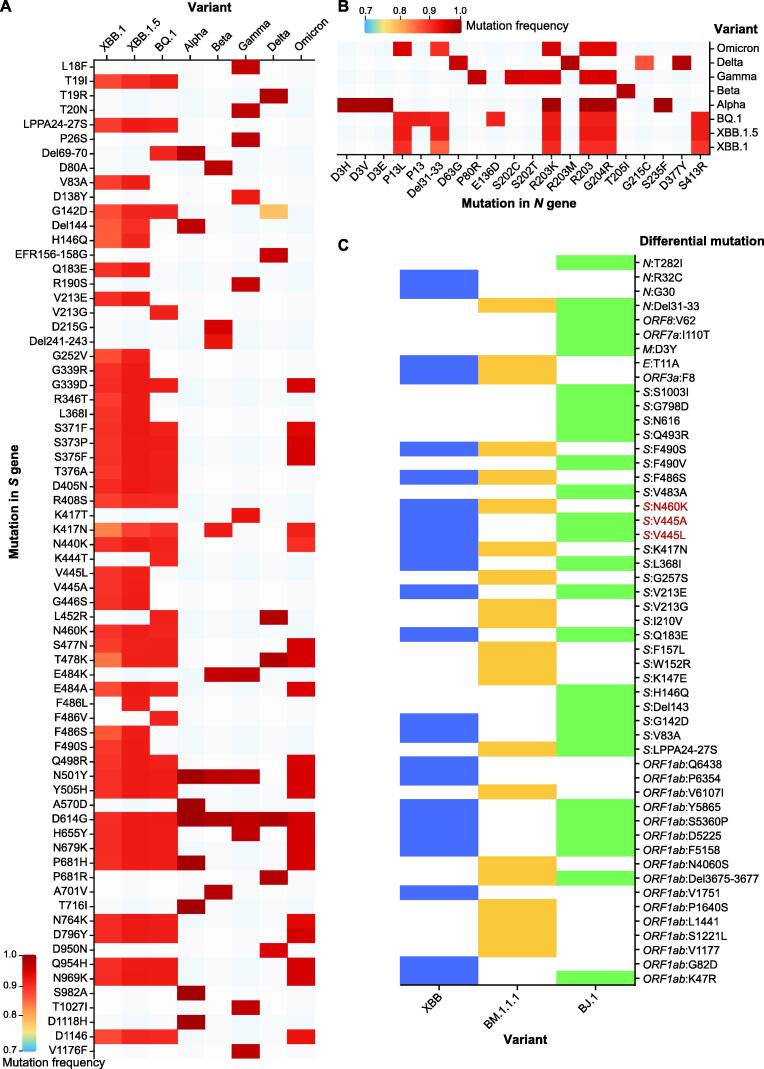
**Mutation spectrum comparison among selected lineages and sequences** **A.** Lineage mutation comparison on the *S* gene among top 3 prevalence lineages in the 10th week of 2023 (XBB.1, XBB.1.5, and BQ.1) and previous VOCs defined by WHO (Alpha, Beta, Gamma, Delta, and Omicron) with mutation frequency. **B.** Lineage mutation comparison on the *N* gene among the top 3 prevalent lineages in the 10th week of 2023 (XBB.1, XBB.1.5, and BQ.1) and previous VOCs defined by WHO (Alpha, Beta, Gamma, Delta, and Omicron) with mutation frequency. **C.** Sequence mutation comparison among sequences (XBB: EPI_ISL_15854782; BJ.1: EPI_ISL_14891585; BM.1.1.1: EPI_ISL_14733830) presented with a heatmap by differential mutations (refers to those after removing common mutations among sequences) in each sequence. The mutations in red color represent possible recombination breakpoints. VOC, variant of concern.

In the inter-lineage or variant comparison module (https://ngdc.cncb.ac.cn/ncov/knowledge/compare), users can examine the mutation patterns across WHO-defined variants (*e.g.*, Delta and Omicron) or Pango lineages (*e.g.*, B.1.177 and XBB.1.5) and analyze mutations by genes or mutation frequency. For example, considering the top 3 prevalent lineages in the 10th week of 2023 (XBB.1, XBB.1.5, and BQ.1) and the previous variants of concern (VOCs) defined by WHO (Alpha, Beta, Gamma, Delta, and Omicron), it is evident that these lineages exhibit more mutations in the *S* gene (Figure 6A). Moreover, several novel mutations with high frequencies, such as S371F, T376A, and S477N (frequency > 0.89), in *S* gene, have emerged in XBB.1 and XBB.1.5. Additionally, well-known mutations, such as D614G (known to enhance SARS-CoV-2 infectivity in human lung cells) and N501Y (associated with reduced vaccine protection in Delta), may explain the prevalence of ongoing XBB variants [24]. In addition to the extensively studied *S* gene, *N* gene mutations like R203K and G204R, implicated in increased transmission [25], are commonly observed in the top 3 ongoing lineages (Figure 6B). Notably, the *N* gene mutation P13L, which occurs at a high frequency of 90% in the top 3 ongoing variants, can significantly impair the CD8^+^ T cell epitope (QRNAPRITF), leading to a loss of T cell recognition [26], [27], [28]. Similarly, amino acid deletions from position 31 to 33 in the N protein, with a high frequency of 90% among ongoing lineages, may contribute to improved replication efficacy or breakthrough infections, warranting further investigation in the future.

In the multiple sequence comparison module (https://ngdc.cncb.ac.cn/ncov/variation/sequence/compare), users can sensitively detect potential new lineages by comparing newly released sequences with the representative sequences of the latest lineages in RCoV19. By inputting accession IDs and selecting the lineages of interest, this module will display a mutation matrix for comparison, which can be further refined interactively by genes or differential mutation sites. The mutation matrix can be color-coded based on lineage, sampling date, or location. Additionally, as we known, recombination contributes new variations in virus evolution, and the identification of the events requires complex algorithms and intensive calculations, such as 3SEQ [29], RDP5 [30], RIPPLES [31], RIVET [32], RecombinHunt [33], and VirusRecom [34]. Our sequence comparison module is particularly useful for narrowing down the breakpoint ranges in identified recombinant variants, which are illustrated by unique mutations among each variant. For example, when analyzing the XBB recombinant lineage, comparing it with its parental sequences (BJ.1: EPI_ISL_14891585; BM.1.1.1: EPI_ISL_14733830) reveals that the breakpoint likely lies between V445 and N460 in *S* gene since XBB harbors V445 from BJ.1 and N460 from BM.1.1.1 (Figure 6C). Overall, this module complements existing platforms [35], [36] and aids in assessing the validity of newly assigned lineages. It also empowers users to explore and compare mutation spectra across different lineages and variants, providing valuable insights into the evolution and characteristics of SARS-CoV-2 lineages.

### Investigation of the mutation effects on transmissibility and immune escape

A number of mutations have been confirmed to affect viral characteristics, including pathogenicity, infectivity, transmissibility, and antigenicity [11], [12], [13], [37], [38]. However, the knowledge is scattered across publications and always focuses on one aspect of a mutation or a variant. To facilitate the effective retrieval of mutation functions, we have constructed an integrated knowledgebase by curating information from literature and databases. Specifically, mutation knowledge is recorded and organized according to the impacts of mutations on infectivity/transmissibility and effectiveness to antibodies, drug, and T cell epitopes.

**Figure 7:**
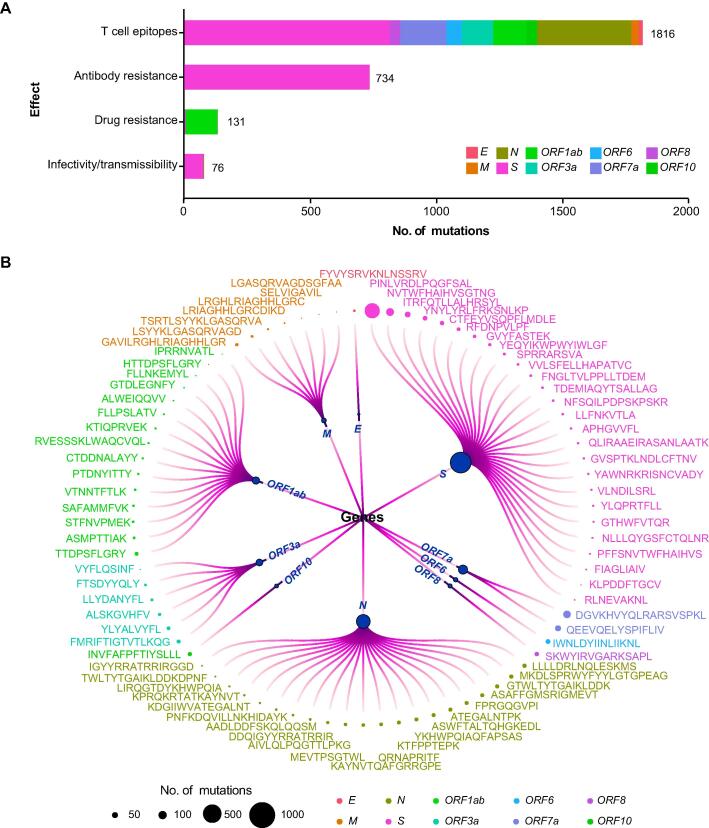
**Mutation effects on SARS-CoV-2 viral characteristics** **A.** Collection of mutation effect knowledge. The X-axis represents the number of mutations. **B.** Mutations occurring in the regions encoding experimentally verified T cell epitopes. The magnitude of the circles represents the number of mutations occurring in the regions encoidng each epitope, and different colors indicate T cell epitopes on different proteins encoded by SARS-CoV-2 genome.

Mutation-related information is collected and categorized based on their specific impacts. For each mutation, we have gathered detailed information, including a comprehensive description, experimental methods used for characterization, and corresponding PubMed IDs (PMIDs) for reference. In the case of T cell epitope mutations, information on epitopes, HLA restriction, and corresponding T cell types has also been integrated. Overall, we have collected and summarized a total of 2693 single mutations/Indels, as well as 19 combined mutations. Among these mutations, 76 affect infectivity/transmissibility, 131 are associated with drug resistance, 734 are related to antibody resistance, and 1816 are located in T cell epitopes (Figure 7A). Mutations are distributed unevenly across genes and open reading frames (ORFs). Specifically, in the *S* gene, 73 mutations (4.7%) have been reported to affect infectivity/transmissibility, while 733 mutations (46.7%) are associated with antibody resistance. This is understandable as the receptor-binding domain (RBD) of the S protein is responsible for virus binding to the ACE2 receptor and is a target for neutralizing antibodies. In the *ORF1ab* gene, 127 mutations (48.8%) are related to drug resistance, which may be attributed to *ORF1ab* being the target of most small molecule inhibitors.

Mutations within regions encoding T cell epitopes are of particular concern as they are dispersed across different genes, posing challenges for the immune system to recognize and mount an effective response against various variants. Moreover, mutations in the regions encoding CD4^+^ and CD8^+^ T cell epitopes have the potential to disrupt HLA-peptide binding, leading to immune escape. The regions encoding diverse epitopes within different genes of SARS-CoV-2 exhibit distinct mutation patterns, which need to be carefully considered during the design of epitope-based vaccines (Figure 7B).

By providing a comprehensive and organized knowledgebase, researchers and users can easily access and retrieve information regarding the functional impacts of specific mutations. This integrated resource (https://ngdc.cncb.ac.cn/ncov/knowledge/mutation) enhances our understanding of the effects of mutations on viral characteristics and assists in the development of effective countermeasures against SARS-CoV-2 variants.

## Discussion

RCoV19 has been continuously updated and developed to support precise prevention of COVID-19. As an integrated repository for SARS-CoV-2 genome data, we have addressed various challenges by implementing a one-stop curation pipeline. This pipeline resolves issues such as sequence redundancy across different repositories, cross-linking between resources, and sequence quality evaluation. However, due to the lack of comprehensive clinical phenotype data, conducting in-depth association studies between massive genomic data and clinical outcomes, as well as unraveling the clinical significance of mutations, remains challenging. To enhance our understanding of disease spread and pathogenesis, we urge the collection and integration of clinical phenotype data of infected individuals to create a more comprehensive platform.

Timely monitoring and precise pre-warning based on genomic data are crucial for epidemic prevention. While there are platforms [4], [5], [6] available for spatiotemporal surveillance of mutations and variant evolution, there is a deficiency in platforms specifically focusing on pre-warning of high-risk variants. Recently, various machine learning-based prediction models have been proposed, such as PyR0 [39] and VarEPS [10]. PyR0, a hierarchical Bayesian multinomial logistic regression model, can identify mutations that are likely to increase SARS-CoV-2 fitness [39], while VarEPS evaluates the risk level of mutations and variants based on their transmissibility and affinity to neutralizing antibodies using a random forest model [10]. In RCoV19, we have developed a LightGBM model called HiRiskPredictor [16], which calculates a comprehensive risk score and predicts potential high-risk haplotypes on a weekly basis. In the future, we aim to provide multidimensional pre-warning by combining the strengths of different AI models and features.

Genetic mutation spectra play a critical role in determining the virological characteristics of different virus strains. Sequence comparison remains the primary approach for identifying differences in mutation spectra. Although the Pango dynamic phylogeny-informed nomenclature system has made significant contributions to tracking genetic diversity and classifying SARS-CoV-2 lineages, there is often a time gap before sporadic variants occurring in specific regions are designated as new lineages. To stay updated on SARS-CoV-2 mutations more sensitively and identify novel lineages earlier, RCoV19 now supports the comparison of newly released sequences with representative sequences of the latest lineages. This feature complements existing public platforms [35], [36] and assists in verifying the assigned lineages of newly released sequences.

Numerous mutations have been identified that they can increase the severity of infections, enhance transmissibility, and enable evasion of natural and vaccine-induced immunity [40]. Through comprehensive literature curation, we have consolidated a wealth of knowledge regarding the effects of mutations on viral infectivity, resistance to antibodies and therapeutic drugs, and alterations to T cell epitopes. However, further investigation is needed on mutations that impact disease severity. For example, the mutation S194L in the *N* gene has a notably high frequency among individuals with severe clinical manifestations [41], suggesting its potential contribution to disease progression. Meanwhile, most of the knowledge on mutation effects is curated from published literature or databases. Future improvements could focus on structural bioinformatics-based prediction of mutation effects, which would enhance our understanding of future pandemics and aid in the development of preventive measures and treatment strategies. In conclusion, knowledge of mutation effects is essential for effective public health interventions, the development of therapeutics, and the generation of pre-warning models. Additionally, automatic gathering of mutation effect information may soon become necessary, *e.g.*, with automatic processes such as ViMRT [42] or crowd-sourcing-based methods such as CoVEffect [43]. However, ViMRT does not classify effects; instead, it extracts phenotype descriptions using regular expression-based methods. In contrast, CoVEffect primarily focuses on training abstracts and has lower support for full texts and tables within the full texts. Therefore, building upon the aforementioned studies, we will aim to develop new algorithms to automate knowledge formation.

## Method

### Pre-warning of potential high-risk haplotypes

All complete and high-quality SARS-CoV-2 sequences and metadata in RCoV19 were used to predict potential high-risk haplotypes weekly. First, we calculated the population mutation frequency (PMF) for each mutated site within every month. Then, those non-UTR mutations with PMF > 0.005 were selected for haplotype network construction by McAN [17] with default parameters. Next, the result of the haplotype network was loaded into HiRiskPredictor with a pre-trained machine learning algorithm (LightGBM) to perform the forewarning analysis process. The HiRiskPredictor automatically extracts features, such as out degree, geographic information entropy, and betweenness, for each haplotype in the network. And HiRiskPredictor infers a risk score indicating the likelihood of a haplotype being positive or classified as high-risk. If the predicted risk score of a haplotype is greater than 0.5, it is defined as a high-risk haplotype.

### Mutation spectrum comparison between selected lineages or sequences

Only complete and high-quality genome sequences were used for downstream analyses, including mutation identification, lineage identification, and mutation spectrum comparison. First, genome sequence alignment was performed with MUSCLE (v3.8.31) [44] by comparing against the earliest released SARS-CoV-2 genome (GenBank: MN908947.3). Sequence mutations were identified directly using an in-house Perl program. Then, we utilized the Pangolin [45] software to predict the Pango lineage of the genome sequence. The software is periodically updated in accordance with its official website. Next, the mutation spectrum comparison module was developed at both the lineage and sequence levels, using the sequence mutation and lineage data. At the sequence level, we selected one of the earliest high-quality genome sequences as the representative sequence for each lineage that has been widely distributed globally. By utilizing these representative sequences as references, users can input the sequence IDs to be compared into the input box and visualize the amino acid differences between these sequences in the form of heatmaps. For the comparison at the lineage level, if a mutation occurs in more than 70% (the frequency cutoff is user defined) of all sequences within a lineage, we identify it as a common mutation of that lineage. By selecting the lineages of interest from the dropdown menu, one can observe the amino acid differences in common mutations among these lineages in the form of heatmaps.

### Investigation of the mutation effects on transmissibility and immune escape

Through a comprehensive literature curation, we collected a curated list of epitopes that have been experimentally validated. These experiments involved interferon-γ (IFN-γ) enzyme-linked immunospot (ELISpot) assays, complex class I (pMHCI) tetramer staining, and peptide-stimulated activation-induced marker (AIM) assays, *etc*. Subsequently, we employed an in-house program to integrate all available mutations within the regions encoding those effective epitopes and filter mutations with sequence count lower than 2000. Then, we conducted a more precise literature curation to search for mutation effect occurring on epitopes to illustrate their functions in T cell recognitions.

## Data availability

The complete contents of RCoV19 are available at https://ngdc.cncb.ac.cn/ncov/.

## Competing interests

The authors have declared no competing interests.

## CRediT authorship contribution statement

**Cuiping Li:** Methodology, Formal analysis, Writing – original draft. **Lina Ma:** Data curation, Methodology, Writing – original draft. **Dong Zou:** Software. **Rongqin Zhang:** Data curation, Methodology, Writing – original draft. **Xue Bai:** Data curation, Writing – original draft. **Lun Li:** Methodology. **Gangao Wu:** Data curation. **Tianhao Huang:** Data curation. **Wei Zhao:** Data curation. **Enhui Jin:** Data curation. **Yiming Bao:** Conceptualization, Supervision, Writing – review & editing. **Shuhui Song:** Conceptualization, Methodology, Writing – review & editing. All authors have read and approved the final manuscript.

## Supplementary material

Supplementary Figure S1 **SARS-CoV-2 genome sequence overlaps among different sources (as of August 16, 2023)** Overlaps are computed by means of either the metadata (isolate name, sampling date, location), or both metadata and sequence.

Supplementary Table S1 **Comparisons among several representative SARS-CoV-2 resources**
