# Supplementary figures and images for: RCoV19: A One-stop Hub for SARS-CoV-2 Genome Data Integration, Variant Monitoring, and Risk Pre-warning
Source: Genomics Proteomics Bioinformatics. 2023 Oct 26;21(5):1066–79. doi: 10.1016/j.gpb.2023.10.004 (PMC10928372; doi:10.1016/j.gpb.2023.10.004)

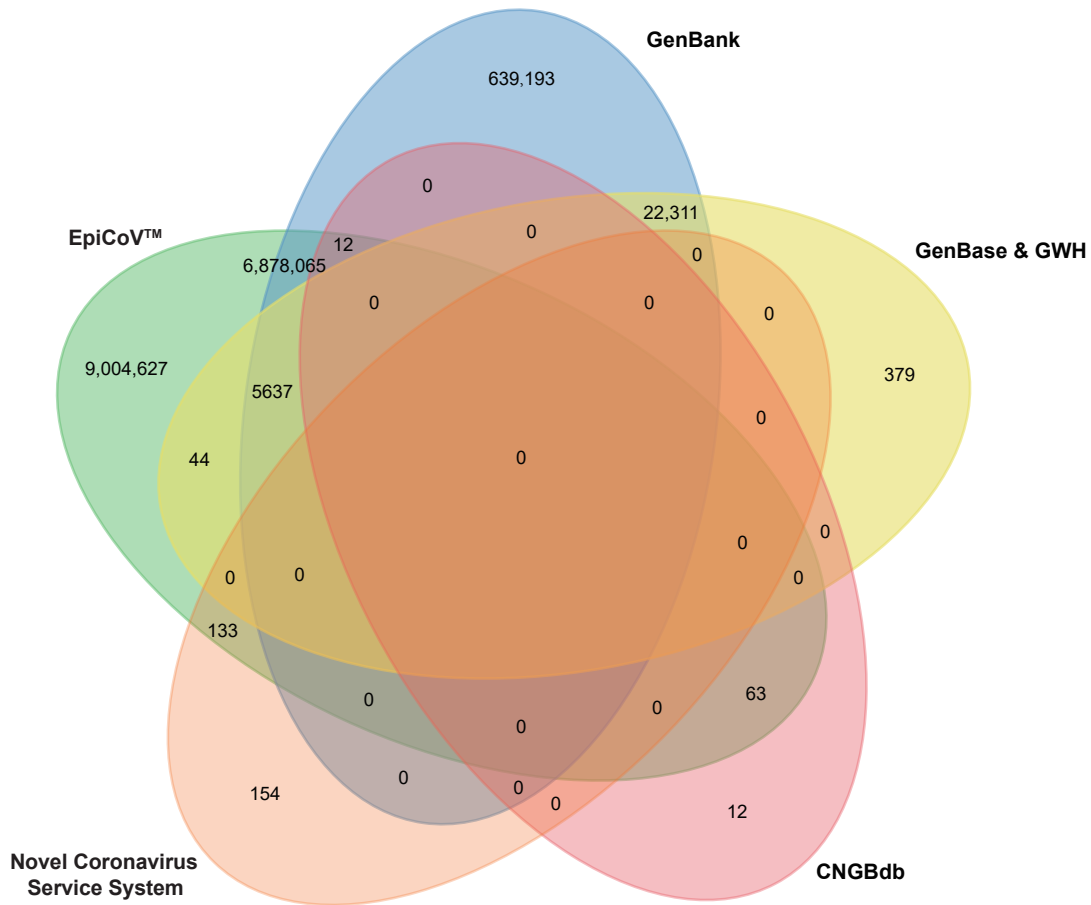

Supplement: Supplementary Figure S1 — SARS-CoV-2 genome sequence overlaps among different sources (as of August 16, 2023) Overlaps are computed by means of either the metadata (isolate name, sampling date, location), or both metadata and sequence. [file mmc1.pdf]
